# Molecular characterization and serodiagnostic potential of a novel dithiol glutaredoxin 1 from *Echinococcus granulosus*

**DOI:** 10.1186/s13071-016-1741-9

**Published:** 2016-08-17

**Authors:** Xingju Song, Min Yan, Dandan Hu, Yu Wang, Ning Wang, Xiaobin Gu, Xuerong Peng, Guangyou Yang

**Affiliations:** 1 Department of Parasitology, College of Veterinary Medicine, Sichuan Agricultural University, Chengdu, China; 2 Department of Chemistry, College of Life and Basic Science, Sichuan Agricultural University, Ya’an, China

**Keywords:** *Echinococcus granulosus*, Cystic echinococcosis, Glutaredoxin, Immunolocalization, Indirect ELISA

## Abstract

**Background:**

The larval stage of *Echinococcus granulosus* is the etiological agent of cystic echinococcosis (CE), which causes serious morbidity and mortality in many areas. There is no reliable method to monitor sheep CE. Here, we characterize *E. granulosus* glutaredoxin 1 (Eg-Grx1) and report an improved immunodiagnostic method for CE.

**Methods:**

We cloned and expressed recombinant Eg-Grx1 and generated antibodies. We analyzed the location of the protein in different parasite stages by fluorescence immunohistochemistry, detected the immunogenicity of recombinant Eg-Grx1, and developed an indirect ELISA (iELISA) for CE serodiagnosis.

**Results:**

Eg-Grx1 is a classic dithiol Grx with several GSH-binding motifs. Native Eg-Grx1 protein was distributed in the tegument of protoscoleces, the whole germinal layer, and the parenchymatous tissue of adult worms. Recombinant Eg-Grx1 exhibited good immunoreactivity to CE-infected sheep serum. An iELISA using this antigen showed specificity of 64.3 % (9/14) and sensitivity of 1:3200, and the diagnostic accordance rate was 97.9 % (47/48) compared with the results of necropsy.

**Conclusion:**

We characterized a novel Grx (Eg-Grx1) from a parasitic helminth and present a comprehensive analysis of the sequence and structure of this protein. The recombinant Eg-Grx1 protein showed good potential serodiagnostic performance, and we established an iELISA method, which may contribute to the surveillance of sheep CE in epidemic areas.

## Background

*Echinococcus granulosus* is a cestode parasite whose larval stage causes cystic echinococcosis (CE) in humans and animals [1]; most cysts (> 90 %) develop in the liver, lungs, or both [2]. CE is a global public health problem causing morbidity and mortality, especially in pastoral areas [3, 4]. Meanwhile, it has been estimated that several billion US dollars are lost annually in the livestock industry as a result of CE [5]. Considering the importance of this disease, the World Health Organization (WHO) has included CE in the list of Neglected Tropical Diseases in its strategic plan [6, 7].

Currently, detection and surveillance of *E. granulosus* infection in livestock relies on necropsy and macroscopic observation procedures in abattoirs [3, 8]. However, these detections without histological examination have a high error rate (15.4 %) [9]. Thus, it is important to establish an inexpensive, accurate immunodiagnostic assay as a surveillance tool for the detection of CE in live animals [10]. Currently, data are limited on recombinant diagnostic antigens for detection of *E. granulosus* infection in sheep [11, 12], and the diagnostic sensitivity of the recombinant proteins used was very low in these reports. Many methods, such as the flow through technique, enzyme linked immuno electrotransfer blot [13, 14], enzyme-linked immunosorbent assay (ELISA) [15], counter-immunoelectrophoresis, and the latex agglutination test [14] have been developed for the diagnosis of CE in sheep. However, these assays use natural *E. granulosus* hydatid cyst fluid antigens, which are difficult and expensive to prepare, and cannot be commercialized. Indirect ELISA (iELISA) using recombinant protein as the antigen has the advantages of high reproducibility and antigen source stability. In consequence, the development of a new recombinant antigen with high diagnostic sensitivity and specificity is a crucial task to improve the immunodiagnosis of CE [12].

Glutaredoxins (Grxs) are ubiquitous oxidoreductases occurring in all organisms and belonging to the thioredoxin family [16]. Grxs maintain the cellular redox equilibrium and catalyze thiol-disulfide exchange reactions by utilizing electrons from the tripeptide glutathione (^γ^Glu-Cys-Gly; GSH) [17–19]. Meanwhile, Grxs can bind to Fe-S clusters, which are involved in intracellular iron sensing, enzyme catalysis, electron transfer and regulation [20–22]. Only a few Grxs from parasites have been reported. *Plasmodium falciparum* Grx1 (PfGrx1) was demonstrated to have characteristics of β-hydroxyethyl disulfide (HEDS) activity, high stability, and resistance to denaturants and pH change [23, 24]. The atomic resolution crystal structure of PfGrx1 was solved and compared with other Grxs [22]. Functional studies on a dithiolic Grx from *Trypanosoma brucei* (TbGrx) suggested that the protein was an important component of the cellular redox metabolism and seemed to be involved in a mechanism to regulate enzymes by glutathionylation/deglutathionylation [25]. Subsequently, a novel report proposed the participation of Grx in redox signaling pathways in *Trypanosoma cruzi*, probably through glutathionylation-deglutathionylation mechanisms [26].

Given that no information on Grxs of parasitic helminthes is available to date, herein, the characteristics of Grx1 from *E. granulosus* (Eg-Grx1) were described. We analyzed the location of this protein in different stages of the parasite, detected the immunogenicity of recombinant Eg-Grx1, and further developed an iELISA assay for the serodiagnosis of CE in sheep. These efforts will contribute to further understanding the characteristics of Eg-Grx1 and improve the diagnosis of this damaging parasitic infection.

## Methods

### Parasites and animals

Cysts of *E. granulosus* were obtained from a slaughterhouse in Qinghai Province, China. The protoscoleces (PSCs) were separated under sterile conditions and washed three times in phosphate-buffered saline (PBS). A 2-month-old dog was infected artificially with 20,000 PSCs. After 35 days, adult worms were collected from the small intestine of the dog. The dog and two female New Zealand white rabbits were obtained from the Laboratory Animal Center of Sichuan Agricultural University. All animals were provided with food pellets and sterilized water *ad libitum*.

### Bioinformatics analysis

The complete gene sequence of Eg-Grx1 (EgrG_000124800) was downloaded from GeneDB (http://www.genedb.org/Homepage). The ExPASy Proteomics Server (http://expasy.org/) was used to predict conserved domains, and the molecular weight and isoelectric point of Eg-Grx1. Amino acid sequence alignment was performed using Clustal X software version 1.83 [27]. A phylogenetic tree was constructed by the neighbor-joining method with MEGA software (version 5.05) [28]. Three-dimensional structural modeling was performed using the Swiss-Model server (http://swissmodel.expasy.org), and the model was based on the crystal structure of *Homo sapiens* Grx1 (PDB accession no: 4RQR) which has a resolution of 1.08 Å [29].

### Expression and purification of recombinant Eg-Grx1

The full coding sequence of Eg-Grx1 was amplified from *E. granulosus* cDNA using primers 5ʹ-CCG GAA TTC ATG TGG CGC TTT TTA TC-3ʹ and 5ʹ-CCG CTC GAG CTC TAA AAG TTC AGC AAG TG-3ʹ, and then integrated into the *Eco*RI/*Xho*I restriction sites of vector pET28a (Novagen, Heidelberg, Germany). Recombinant protein was expressed and purified as previously described [30]. Briefly, the plasmid was transformed into *Escherichia coli* BL21 (DE3) cells (Cowin Biotech, Beijing, China) and induced with 0.8 mM isopropyl-1-thio-β-D-galactopyranoside at 37 °C for 6 h. Proteins were then purified using a Ni^2+^ affinity column (Bio-Rad, Hercules, CA) following the manufacturer’s instructions. The purified rEg-Grx1 protein was monitored by 15 % SDS-PAGE, and the protein concentration was estimated with a bicinchoninic acid protein assay kit (Pierce, Rockford, IL).

### Sera

Twenty-four positive sera against *E. granulosus* were isolated from naturally infected sheep. Negative sera (48 samples) were collected from 48 healthy sheep (confirmed by autopsy). Goat serum positive against C*ysticercus tenuicollis* (7 samples) and sheep serum positive against *Coenurus cerebralis* (7 samples) were also obtained. All sera were from animals from Sichuan Province, China. Polyclonal antibody against rEg-Grx1 was produced as previously described [30]. Briefly, each rabbit was immunized subcutaneously with rEg-Grx1 emulsified in Freund’s complete adjuvant (Sigma, St Louis, MO, USA) with three boosters. A substitution of Freund’s incomplete adjuvant for Freund’s complete adjuvant was used in the final vaccination. After 2 weeks, rabbit anti-rEg-Grx1 serum was detected by ELISA and purified using HiTrap Protein A affinity chromatography (Bio-Rad).

### Western blotting

For immunoblotting, rEg-Grx1 and total PSCs extracts were separated by 15 % SDS-PAGE and then electrotransferred onto a nitrocellulose (NC) membrane. The membranes were incubated with 5 % (w/v) skim milk for 1 h at room temperature, followed by incubation with sheep anti-*E. granulosus* serum or rabbit anti-rEg-Grx1 IgG (1:200 v/v dilution) overnight at 4 °C. Subsequently, horseradish peroxidase (HRP)-conjugated goat anti-rabbit IgG or rabbit anti-sheep IgG (Bio-Rad) and the Enhanced HRP-DAB Chromogenic Substrate Kit (Tiangen, Beijing, China) were used to visualize reactions.

### Immunolocalization of Eg-Grx1 protein in *E. granulosus*

For immunolocalization studies, fresh PSCs, germinal layers and adult worms were embedded in paraffin wax. All sections were dewaxed in xylene and dehydrated in an ethanol series, followed by treatment in 0.01 M citrate buffer (pH 6.0) at 95 °C for 15 min. Subsequently, the sections were incubated with 5 % BSA for 1 h at 37 °C and anti-rEg-Grx1 rabbit IgG (1:200) overnight at 4 °C. After four washes, the sections were reacted with fluorescein isothiocyanate (FITC)-conjugated goat anti-rabbit IgG (H + L) (1:200 dilution in 1 % Evans Blue; Bethyl Laboratories, Montgomery, TX) for 1 h at 37 °C in darkness and then imaged by fluorescence microscope (Nikon, Tokyo, Japan). The serum from preimmune rabbit was used as the negative control.

### ELISA procedure

ELISAs were performed essentially as described [31, 32]. Briefly, the optimal concentration of rEg-Grx1 antigen and serum was assessed by standard checkerboard titration procedures. The purified Eg-Grx1 protein was serially two-fold diluted to six different concentrations (ranging from 6.4 to 0.2 μg/well) in 0.1 M carbonate buffer (pH 9.6) and used as antigen in the iELISA. The ELISA plates were coated with diluted antigen solution overnight at 4 °C. After washing with phosphate buffered saline-Tween-20 (PBST), the plates were incubated with 5 % skim milk for 1 h at 37 °C. The wells were washed thoroughly and incubated with 100 μl of serum samples with twofold dilutions (1:20, 1:40, 1:80, 1:160, 1:320, 1:640) in PBS at 37 °C for 1.5 h. Following washing procedures, 1:200 dilutions of HRP-labeled rabbit anti-sheep or goat IgG (Boster Bio-project Co., Wuhan, China) were added to the plates, which were then incubated at 37 °C for 1 h. Then, the wells were washed again and incubated with the substrate TMB (Tiangen, Beijing, China) at 37 °C for 20 min. Finally, color development was stopped with 100 μl of 2 M H_2_SO_4_, and the optical density at 450 nm (OD_450_) was recorded using a microplate reader (Thermo Scientific, Pittsburgh, PA). Other optimal conditions were explored as in previous reports [33]. An OD_450_ value of positive serum close to 1.0 and the highest P/N value between positive and negative serum were regarded as optimal. The cut-off value of the iELISA was determined by testing 24 negative serum samples from healthy sheep and was calculated as the mean OD_450_ plus three standard deviations of the OD_450_.

### Specificity and sensitivity of iELISA

Serum samples positive for *C. cerebralis* (*n* = 7) and *C. tenuicollis* (*n* = 7) were used to evaluate the cross-reactivity (specificity) of the iELISA. The percentage specificity was calculated as indirect ELISA negative × 100/true negative. Three *E. granulosus*-positive sheep serum samples were used to assess the sensitivity of the iELISA with twofold serial dilutions ranging from 1:100 to 1:25,600.

### Clinical testing of the iELISA

The reliability of the iELISA was evaluated using 48 sheep serum samples, including 24 positive sera and 24 negative sera. The positive diagnostic rate was calculated based on the cut-off value. Each serum sample was tested three times.

### Repeatability and reproducibility of iELISA

The inter-assay variation (between plates) and the intra-assay variation (within a plate) were evaluated by the coefficient of variation (CV) with six positive serum samples. Every sample was detected in three plates to assess the inter-assay CV, and three replicates within each plate were used to assess the intra-assay CV.

### Statistical analysis

All data are presented as the mean ± SD. Statistical analyses were performed by *t*-test and one-way ANOVA for comparison between groups using the software package GraphPad Prism (www.graphpad.com). *P*-values < 0.05 were considered to be significant.

## Results

### Sequence characterization and phylogenetic analysis of Eg-Grx1

**Fig. 1:**
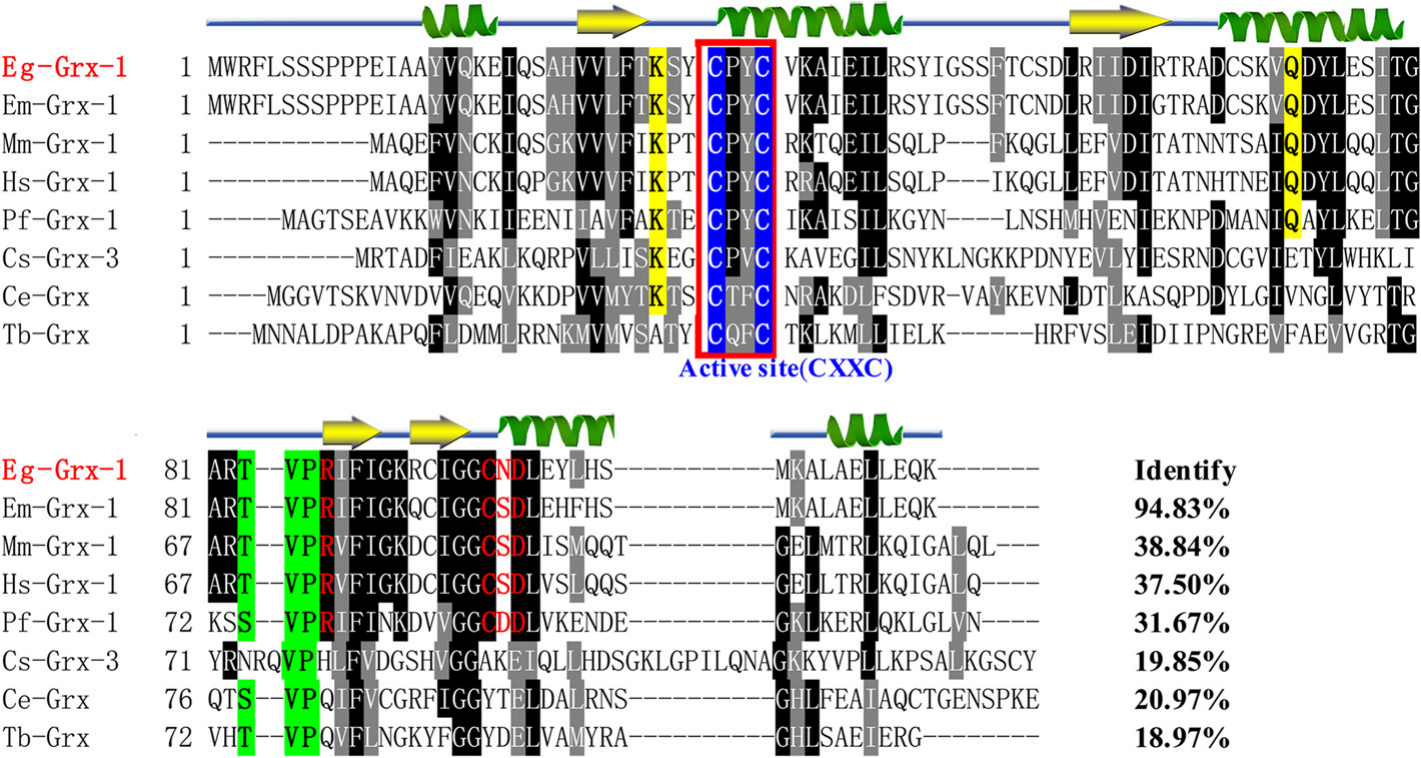
Sequence alignment analysis of Eg-Grx1 and homologous glutaredoxins. The percentage homology of Eg-Grx1 with each glutaredoxin is shown at the end of the alignment. Regions of high identity and similarity between dithiol glutaredoxin sequences are shown as *black* and *gray* columns, respectively. The active-site residues CXXC are marked with a *red* box. The residues involved in interactions with the Gly and Cys of GSH are highlighted with *yellow* and *green* backgrounds. The residues interacting with ^γ^Glu of GSH are indicated by red letters. The predicted secondary structure of Eg-Grx1 is displayed above the alignment. Database accession numbers: *Echinococcus granulosus* glutaredoxin 1 (Eg-Grx-1) (GeneDB: EgrG_000124800), *Echinococcus multilocularis* glutaredoxin 1 (Em-Grx-1) (GenBank: CDI97471.1), *Mus musculus* glutaredoxin 1 (Mm-Grx-1) (GenBank: NP_444338.2), *Homo sapiens* glutaredoxin 1 (Hs-Grx-1) (GenBank: AAC35798.1), *Plasmodium falciparum* glutaredoxin 1 (Pf-Grx-1) (GenBank: BAB79691.1), *Clonorchis sinensis* glutaredoxin 3 (Cs-Grx-3) (GenBank: GAA51070.1), *Caenorhabditis elegans* glutaredoxin (Ce-Grx) (GenBank: NP_001040891.1), and *Trypanosoma brucei* glutaredoxin (Tb-Grx) (GenBank: CBV36792.1)

**Fig. 2:**
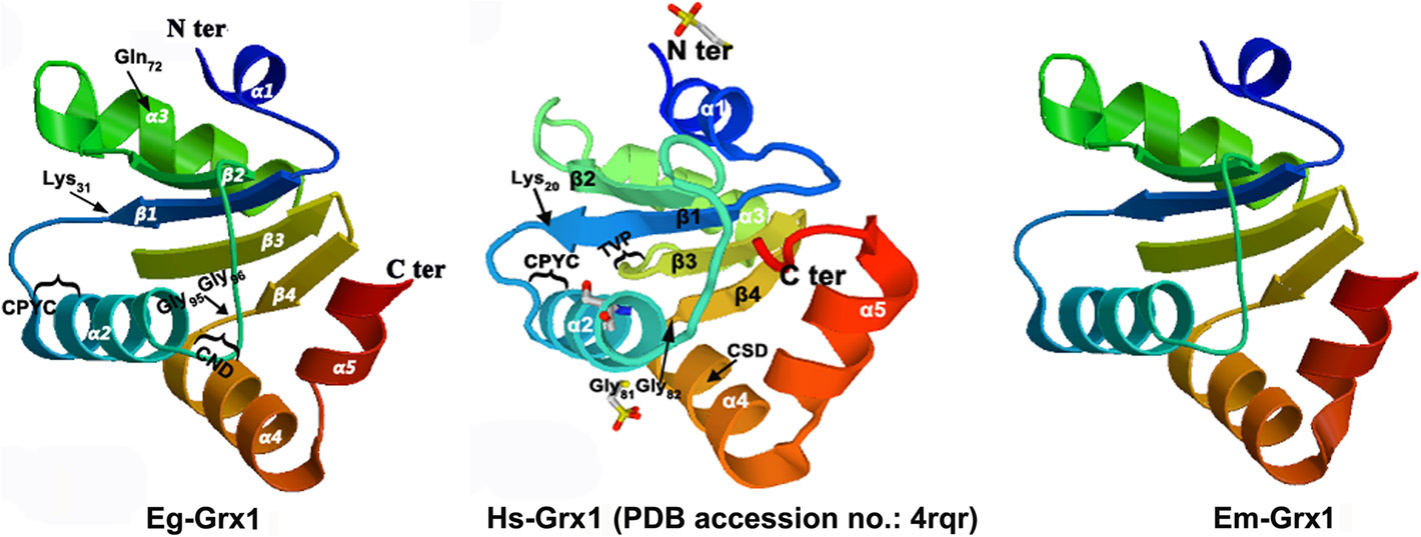
Three-dimensional structural of Eg-Grx1 and homologous proteins from *Homo sapiens* and *Echinococcus multilocularis*. The 3D structures were built based on the crystal structure of *Homo sapiens* Grx1 (Hs-Grx1) (PDB accession code 4RQR). The secondary structural elements and active-site residues (Lys and Gln, CPYC, TVP, Gly-Gly, CS/ND) are displayed on the 3D structures

The full-length cDNA of the Eg-Grx1 gene contained a single ORF of 351 bp encoding 116 amino acids. The predicted molecular mass and pI were 13.2 kDa and 8.8, respectively. No signal peptide or transmembrane regions were predicted. Sequence analysis showed that Eg-Grx1 had a Cys-Pro-Tyr-Cys (CPYC) active site, which is a classic dithiol motif [34]. Multiple sequence alignment revealed the highest similarity (94.83 %) between Eg-Grx1 and *Echinococcus multilocularis* Grx1 (Em-Grx1). Eg-Grx1 showed 31.67–38.84 % identity with glutaredoxin 1 from *Mus musculus* (Mm-Grx1), *Homo sapiens* (Hs-Grx1) and *Plasmodium falciparum* (Pf-Grx1) (Fig. 1). Sequence analysis also revealed that GSH-binding motifs (CXXC, Lys and Gln/Arg, TVP, and CXD) were conserved in Eg-Grx1 (Fig. 1); a conserved Gly-Gly motif was also found. The three-dimensional structure of Eg-Grx1 was conserved (judged by structural modeling) and included a β-sheet of four strands surrounded by three α-helices (Fig. 2).

**Fig. 3:**
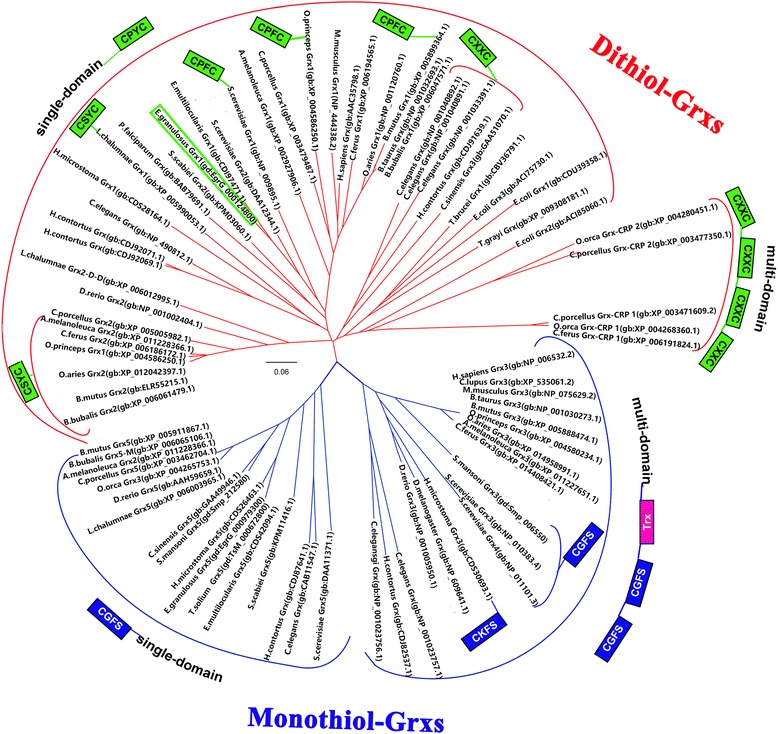
Phylogenetic tree of Eg-Grx1 and Grxs from other species. The tree was constructed using the neighbor-joining method in MEGA software version 5. The glutaredoxin domain is indicated by *green*, *blue* or *pink* boxes. *Abbreviations*: Trx, thioredoxin-like; gd, GeneDB; gb, GenBank

The relationship between various Grx sequences is displayed in a phylogenic tree (Fig. 3), which showed that Grxs divided into two clades. One consisted of the dithiol Grxs with a CXXC consensus active site, and the other of the monothiol Grxs with a CXXS consensus active site. Eg-Grx1 clustered into the dithiol Grx clade.

### Expression, purification and identification of recombinant Eg-Grx1 (rEg-Grx1)

**Fig. 4:**
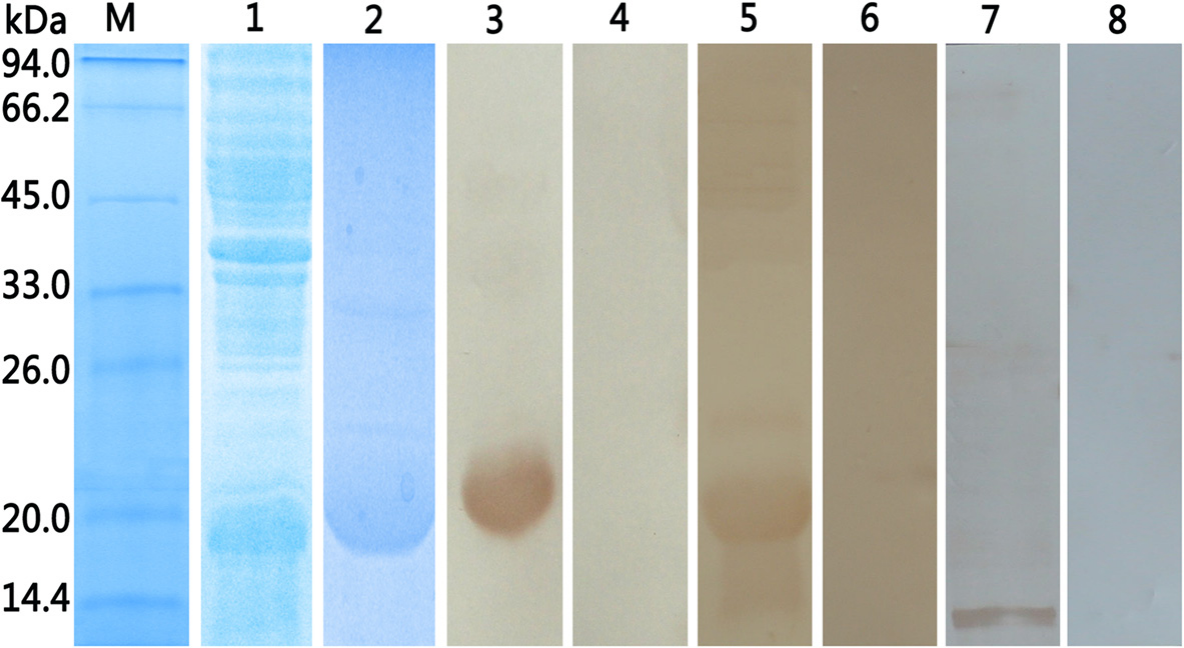
SDS-PAGE and western blotting analysis of Eg-Grx1. Lane M, molecular weight marker; Lane 1, IPTG-induced *E. coli* BL21 (DE3) lysate from cells expressing rEg-Grx1; Lane 2, purified rEg-Grx1; Lane 3, purified rEg-Grx1 probed with anti-rEg-Grx1 rabbit serum; Lane 4, purified rEg-Grx1 probed with native (pre-immune) rabbit serum; Lane 5, purified rEg-Grx1 probed with the serum of *E. granulosus* infected sheep; Lane 6, purified rEg-Grx1 probed with native (healthy) sheep serum; Lane 7, the total protein from protoscoleces probed with anti-Eg-Grx1 rabbit serum; Lane 8, the total protein from protoscoleces probed with native (pre-immune) rabbit serum

Recombinant Eg-Grx1 was expressed with a His-tag. The purified rEg-Grx1 protein showed a single band on 15 % SDS-PAGE, around the expected size of 19 kDa (including the His-tag) (Fig. 4). In western blotting, the native Eg-Grx1 protein from PSC extract could be recognized using anti-rEg-Grx1 rabbit IgG, and its apparent molecular mass was, as expected, ~13 kDa. Recombinant Eg-Grx1 was probed with anti-rEg-Grx1 rabbit IgG (positive control) and serum from sheep infected with *E. granulosus* (experimental control). A single band of ~19 kDa was observed on the NC membrane, while no band was observed in the negative control (Fig. 4).

### Fluorescence immunohistochemical analysis

**Fig. 5:**
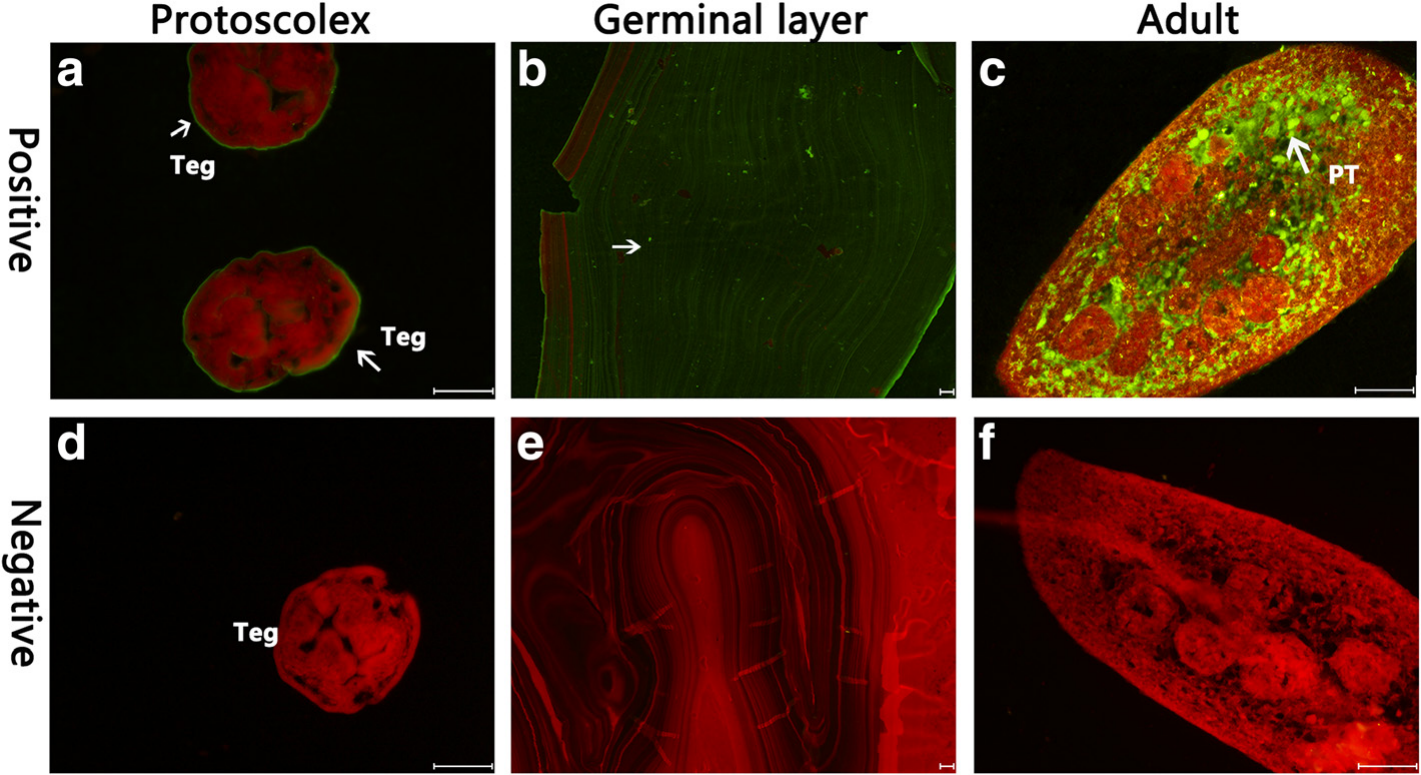
Immunofluorescence localization of Eg-Grx1 in different stages of *E. granulosus*. Eg-Grx1 was localized on the protoscolex (**a**), germinal layer (**b**) and adult (**c**) using specific anti-rEg-Grx1 IgG (positive), or control preimmune serum (negative) (**d**, **e** and **f**, respectively). *Abbreviations*: Teg, tegument; PT, parenchymatous tissue. *Arrows* indicate: **a** positive signal in tegument (Teg); **b** positive signal in germinal layer; **c** positive signal pinparenchymatous tissue (PT). *Scale-bars*: 50 μm

Native Eg-Grx1 was distributed in the tegument of PSCs, and widely distributed in the whole germinal layer. In adult worms, strong fluorescence signals were observed in the parenchymatous tissue (Fig. 5). No signal was detected in the negative controls.

### ELISA

**Fig. 6:**
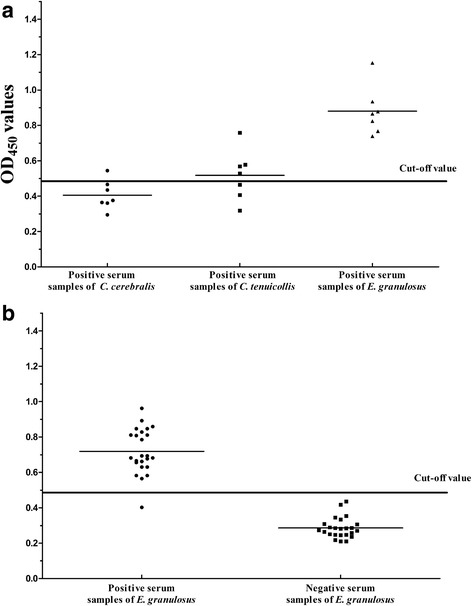
iELISA for the detection of cystic echinococcosis in sheep. **a** The specificity of the iELISA. Statistically significant differences between *E. granulosus*-positive sera and the the other positive sera were tested by one-way ANOVA using GraphPad Prism software (ANOVA: F_(2, 18)_ = 28.41, *P* < 0.0001). No difference was noted between the the *C. cerebralis*-positive and *C. tenuicollis*-positive sera samples. **b** Clinical trial of the iELISA. A statistically significant difference between the *E. granulosus*-positive group and the negative group was determined by *t*-test (t_(46)_ = 15.12, *P* < 0.0001). The cut-off value was 0.481

To establish the iELISA method, we first determined that the optimal antigen concentration and serum dilution were 1.6 μg/well and 1:320, respectively. The cut-off value determined using negative serum was 0.481. Positive serum samples from *C. tenuicollis*-infected goats and *C. cerebralis*-infected sheep were tested to evaluate the cross-reactivity of this iELISA. Four *C. tenuicollis*-positive serum samples (*n* = 7) and one *C. cerebralis*-infected serum sample (*n* = 7) cross-reacted with rEg-Grx1, corresponding to an overall specificity of 64.3 % (9/14) (Fig. 6a). There were statistically significant differences observed in the ELISA values between the *E. granulosus*-positive sera and the other positive sera (ANOVA: F_(2, 18)_ = 28.41, *P* < 0.0001). No difference was noted between the *C. cerebralis*-positive and *C. tenuicollis*-positive sera samples. Based on the cut-off value of this method, we found that the minimum detection limit of positive serum samples was a dilution of 1:3200 (mean absorbance measurement = 0.527).

Forty-eight sheep serum samples (24 positive, 24 negative) were used to evaluate the reliability of our iELISA method. The ELISA recognized 23 samples from the infected sheep as positive, and the remaining samples were found to be negative. The compliance rate was 97.9 % (47/48) compared with the results of necropsy (Fig. 6b). A significant difference was observed between the positive group and the negative group (t_(46)_ =15.12, *P* < 0.0001).

As for the reproducibility and repeatability of our iELISA method, the inter-assay CVs ranged from 0.914 to 1.558 % (mean 1.153 %), while the intra-assay CVs ranged from 0.583 to 2.014 % (mean 1.191 %). These data showed that the coefficients were < 10 %, which means that this assay was repeatable and reproducible.

## Discussion

Grxs and thioredoxins are important members of enzymatic and non-enzymatic antioxidant systems, respectively. Grxs use the reducing power of glutathione to play crucial roles in the redox homeostasis of the cell and redox-dependent signaling pathways [17, 35]. They have been implicated in various physiological processes, such as immune defense, neurodegeneration, cardiac hypertrophy, and protection of cells from hydrogen peroxide-induced apoptosis [17]. However, knowledge of Grx proteins in parasitic helminths is lacking. This study characterized *E. granulosus* glutaredoxin 1, and tested the recombinant protein as a diagnostic candidate antigen for CE in sheep.

All Grxs have a common thioredoxin fold, containing a four-stranded β-sheet surrounded by three to five α-helices. They can be generally classified as monothiol (CXXS) and dithiol (CXXC) Grxs depending on the number of cysteine residues present in the redox active site [22, 36]. In the monothiol mechanism, only the (single) N-terminal cysteine can be used for reduction of Grx-GSH mixed disulfides, while the dithiol Grxs can use both active-site cysteines [37, 38]. Dithiol Grxs can be further categorized into multi-domain dithiol Grxs consisting of two or four dithiol Grx domains and single-domain dithiol Grxs containing only one dithiol Grx domain. Meanwhile, monothiol Grxs are subdivided into single-domain monothiol Grxs, and multi-domain monothiol Grxs that contain one Trx-like domain and two monothiol Grx domains [17]. Our sequence alignment and phylogenetic analysis showed that Eg-Grx1 had one dithiol motif (CXXC) and was classified as a single-domain dithiol Grx.

Grxs are defined by their ability to bind and utilize GSH as substrate [17]. In this study, conserved motifs including TVP, CXD, Lys and Gln/Arg were found in the Eg-Grx1 sequence, which are thought to be involved in interaction with Cys, ^γ^Glu, and Gly of GSH, respectively [22]. A Gly-Gly motif has also been defined as a Grx-characteristic motif [17]; Eg-Grx1 has this two glycine motif (Gly95-Gly96). With Thr83 of the TVP motif and Tyr36 in the active site, this GG site was reported to form a binding groove for GSH on the protein surface of *T. brucei* Grx1 [39].

Parasites deal with oxidative stress (reactive oxygen species and reactive nitrogen species) from both their own metabolism and the immune system of the host [40–42]. As an antioxidant protein, Eg-Grx1 should play crucial roles in antioxidant processes, and help *E. granulosus* survive [43]. Previous research on *E. coli* and *T. brucei* Grxs showed that the expression levels varied during different growth phases [38, 39]. In *Saccharomyces cerevisiae*, Grx6 localized at the endoplasmic reticulum and Golgi compartments, while Grx7 was localized mostly at the Golgi, and the expression of Grx6 and Grx7 was upregulated by some stresses (such as calcium, sodium and peroxides) [44]. Human Grx3 was normally located in the cytoplasm, but under oxidative stress it could be transferred from the cytoplasm to the nucleus [45]. It is interesting to note that in the present study, Eg-Grx1 was observed in every stage of the parasite, but with a diverse distribution pattern in each life-cycle stage. A possible explanation is that the two developmental stages of *E. granulosus* are parasitic on different hosts and different organs, which might lead to different oxidative stresses. Therefore, to provide protection from damage by ROS, the antioxidant enzyme Eg-Grx1 may be expressed in different locations and at different levels in different stages of *E. granulosus*.

Due to the fact that *E. granulosus*-infected sheep will not show obvious clinical symptoms, it is difficult to detect before slaughter. Some research has contributed to the development of recombinant diagnostic antigens for screening for CE in domesticated animals. However, these studies using recombinant EG95 oncosphere protein and recombinant AgB protein in ELISA exhibited poor sensitivity (1.6 and 28 % for sheep CE, respectively) [11, 12]. Thus, screening a new recombinant antigen with high diagnostic sensitivity and specificity for sheep CE is important. Many antioxidant enzymes, such as glutathione S-transferase [46], thioredoxin peroxidase [47] and superoxide dismutase [48], have been confirmed to be diagnostic antigens for parasites. Here, an iELISA diagnostic method for sheep CE was established based rEg-Grx1. The iELISA could clearly detect *E. granulosus*-specific IgG antibodies, and the compliance rate was 97.9 % (47/48) compared with the results of necropsy. The specificity of the assay was not high (64.3 %). Nevertheless, it is worth noting that the cross-reaction occurred mainly in the detection of *C. tenuicollis*-positive sera, and only one cross-reaction was observed with *C. cerebralis*-positive serum. This cross-reactivity might be due to the migration of both *E. granulosus* and *C. tenuicollis* causing visceral lesions [1, 49], while *C. cerebralis* mainly causes lethal neurological symptoms [32], and thus the level of antibody response generated by the host to the various parasites is different. Although the iELISA method here is a poor in distinguishing *E. granulosus* infection in sheep from *C. tenuicollis* infection in goats, its compliance rate and sensitivity in the diagnosis of CE are high compared with previous reports. Thus, this method can be used in preliminary screening for sheep CE in epidemic areas.

## Conclusions

In this study, we identified Eg-Grx1 as a single-domain dithiol Grx. We present a comprehensive demonstration of the structural characteristics and tissue distributions of Eg-Grx1, providing novel insights into its biological functions. We have also performed a preliminary evaluation of the diagnostic potential of rEg-Grx1 based on an iELISA method, indicating that it could be an efficient antigen for preliminary mass screening of *E. granulosus* infections in sheep in highly epidemic areas. Research using this iELISA in conjunction with sera from sheep infected with other *Taenia* species should be carried out to further assess cross-reactivity.

## Abbreviations

Eg-Grx1: *E. granulosus* Glutaredoxin 1
CE: Cystic echinococcosis
iELISA: Indirect ELISA
Grxs: Glutaredoxins
PSCs: Protoscoleces
NC: Nitrocellulose
CV: Coefficient of variation
